# Pathological findings in organs and tissues of patients with COVID-19: A systematic review

**DOI:** 10.1371/journal.pone.0250708

**Published:** 2021-04-28

**Authors:** Sasha Peiris, Hector Mesa, Agnes Aysola, Juan Manivel, Joao Toledo, Marcio Borges-Sa, Sylvain Aldighieri, Ludovic Reveiz

**Affiliations:** 1 Pan American Health Organization, Health Emergencies Department, Washington, Columbia, United States of America; 2 Pan American Health Organization, Incident Management Systems for COVID-19, Washington, Columbia, United States of America; 3 Department of Pathology and Laboratory Medicine, Indiana University School of Medicine, Indianapolis, Indiana, United States of America; 4 Department of Pathology and Laboratory Medicine, University of Florida College of Medicine, Jacksonville, Florida, United States of America; 5 Department of Pathology and Laboratory Medicine, University of Minnesota and VA Healthcare System, Minneapolis, Minnesota, United States of America; 6 Multidisciplinary Sepsis Unit, Intensive Care Unit, Institute of Health Research of de Balearic Islands (IDISBA), Son Llatzer University Hospital, Palma de Mallorca, Spain; 7 Pan American Health Organization, Evidence and Intelligence for Action in Health Department, Washington, Columbia, United States of America; Maastricht University Medical Center, NETHERLANDS

## Abstract

**Background:**

Coronavirus disease (COVID-19) is the pandemic caused by SARS-CoV-2 that has caused more than 2.2 million deaths worldwide. We summarize the reported pathologic findings on biopsy and autopsy in patients with severe/fatal COVID-19 and documented the presence and/or effect of SARS-CoV-2 in all organs.

**Methods and findings:**

A systematic search of the PubMed, Embase, MedRxiv, Lilacs and Epistemonikos databases from January to August 2020 for all case reports and case series that reported histopathologic findings of COVID-19 infection at autopsy or tissue biopsy was performed. 603 COVID-19 cases from 75 of 451 screened studies met inclusion criteria. The most common pathologic findings were lungs: diffuse alveolar damage (DAD) (92%) and superimposed acute bronchopneumonia (27%); liver: hepatitis (21%), heart: myocarditis (11.4%). Vasculitis was common only in skin biopsies (25%). Microthrombi were described in the placenta (57.9%), lung (38%), kidney (20%), Central Nervous System (CNS) (18%), and gastrointestinal (GI) tract (2%). Injury of endothelial cells was common in the lung (18%) and heart (4%). Hemodynamic changes such as necrosis due to hypoxia/hypoperfusion, edema and congestion were common in kidney (53%), liver (48%), CNS (31%) and GI tract (18%). SARS-CoV-2 viral particles were demonstrated within organ-specific cells in the trachea, lung, liver, large intestine, kidney, CNS either by electron microscopy, immunofluorescence, or immunohistochemistry. Additional tissues were positive by Polymerase Chain Reaction (PCR) tests only. The included studies were from numerous countries, some were not peer reviewed, and some studies were performed by subspecialists, resulting in variable and inconsistent reporting or over statement of the reported findings.

**Conclusions:**

The main pathologic findings of severe/fatal COVID-19 infection are DAD, changes related to coagulopathy and/or hemodynamic compromise. In addition, according to the observed organ damage myocarditis may be associated with sequelae.

## Introduction

On March 11, 2020, the World Health Organization classified the severe acute respiratory syndrome coronavirus 2 (SARS-CoV-2) as a pandemic [1, 2]. COVID-19 affects mainly the respiratory system and the clinical presentation ranges from asymptomatic cases to severe manifestations. In individuals with comorbidities and other yet to be characterized host factors, it can cause severe morbidity and mortality, usually in the form of acute respiratory distress syndrome (ARDS) that may progress to multiorgan failure and death. The death toll of COVID-19 is approximately 898,000 worldwide at the time this manuscript was prepared [1, 3, 4].

COVID-19 pathophysiology resembles that of other coronavirus infections [5, 6]; this involves attachment of the SARS-CoV-2 virus to the angiotensin-converting enzyme 2 (ACE2) on target cells, followed by internalization and replication of the virus. ACE2 receptors are highly expressed in upper and lower respiratory tract cells, which determines the highest concentration of viral particles at these sites and explains the high contagiousness of oronasal droplets and aerosols, and the preponderance of respiratory symptoms [7]. However, ACE2 are expressed to a lesser degree in non-respiratory tissues like myocardial cells, renal epithelial cells, enterocytes, and endothelial cells in multiple organs, which may explain some of the extrapulmonary manifestations.

In addition to presumed direct cytopathic viral injury, severe COVID-19 infection is frequently complicated by an infection induced microangiopathy or hypercoagulable state that causes capillary, venous and/or arterial thrombosis [8], and that may lead to end-organ damage due to distant thrombotic or embolic disease [9]. The course of severe COVID-19 disease can be further complicated by pre-existing comorbidities, superinfection with community-acquired or nosocomial organisms, and ventilator-associated lung injury [4, 10].

For epidemiologic analyses of new or emerging diseases, the study of tissue biopsies and autopsy material is a well-established method for researching pathogenetic mechanisms and determining the effects of disease in various tissues and organs, and the cause of death [11]. Recently published systemic reviews on autopsies addressed the pathophysiological timeline [12] or summarized histopathological changes in different organs [13]. We performed a systematic review of biopsy and autopsy findings by a team of anatomic, hematopathology, coagulation and public health specialists to provide relevant clinical-pathologic correlations that may help guide therapeutic and preventive steps to prevent further deaths.

### Objectives

- To summarize the reported pathologic findings from biopsies and autopsies of severe COVID-19 cases.
- To document the organs in which the SARS-CoV-2 virus has been detected on tissue specific cells.
- To evaluate whether alterations in affected tissues and organs are the result of direct viral cytopathic injury or of immune/inflammatory-mediated abnormalities leading to microangiopathy and coagulopathy.
- Based on the main pathologic findings identified, clinicopathologic correlations will be attempted to help guide the management of patients with severe COVID-19.

## Methods

### Search strategy and databases

A structured search was conducted of Embase (Medline included) from January 2020 to 4 August 2020 and in PubMed, MedRxiv, Lilacs and Epistemonikos from January 2020 to 17 June 2020 by two authors (SP and LR). The search terms included MeSH terms and key words in the title, abstract and/or as key words (see S1 Text) with no restrictions on year published, type of publication or language. Studies not in English were translated using automatic translation tools. Forward citation searches and references of all papers identified by the search for inclusion were also performed.

### Study selection/ Inclusion and Exclusion criteria

Using PRISMA format, the results of the initial search strategy were first screened by title and abstract, duplicates and studies not meeting the inclusion criteria were excluded by two independent authors.

Inclusion criteria: (1) studies reporting pathologic findings at autopsy from patients with proven COVID-19 infection; (2) Studies reporting pathologic tissue findings of biopsies obtained from proven COVID-19 patients; (3) Case reports and case series including pediatric cases. Peer-reviewed and non-peer reviewed publications were included.

Exclusion criteria (1) lack of pathologic data; (2) patients with other SARS-like illnesses; and (3) In vitro experiments, qualitative and modelling studies.

### Data collection process and data items

Data from the full texts were collected using a template with information (excel spreadsheet) by one author (SP). Two other authors (HM and AA) checked the accuracy of the extracted data. Disagreements between the reviewers were resolved by consensus.

### Data extracted

Study characteristics: country, author, type of study, sample size, peer-reviewed or not.

Clinical information: demographics, comorbidities, clinical presentation, laboratory test results (e.g., blood type, biomarkers, liver function tests, coagulation parameters), imaging, treatment, and hospitalization days.

Pathology: gross, microscopic, and related ancillary studies by site, including documentation of the presence/absence of virus in the examined tissue.

### Methodological quality assessment

The quality assessment was conducted on the domains of selection, ascertainment, causality and reporting [14].

### Strategy for data synthesis

A narrative synthesis of the evidence was also undertaken, and summary tables produced per organ system. Meta-analysis was not appropriate as data synthesis was derived from case reports and case series. Ethical approval was not required for this review.

## Results

**Fig 1:**
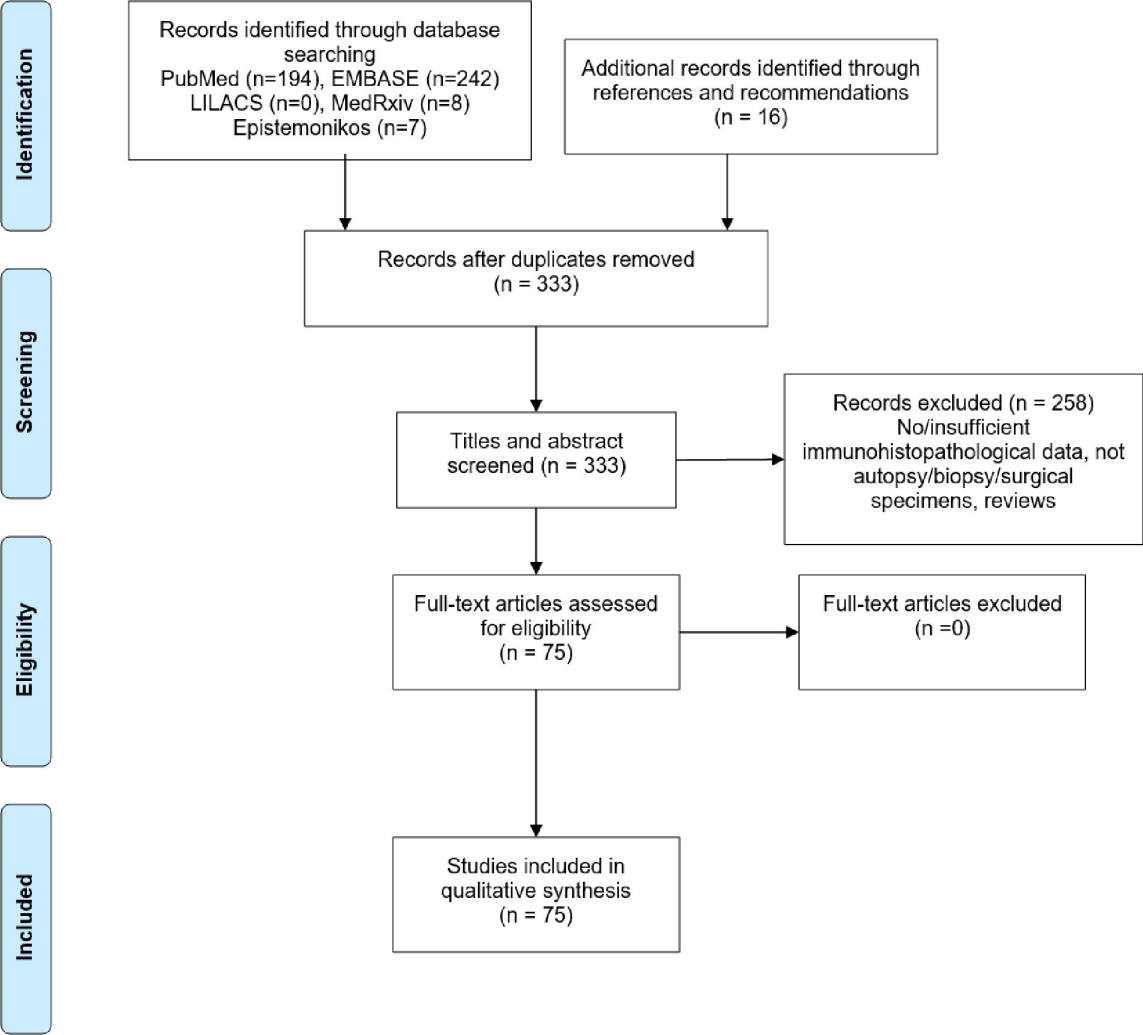
PRISMA flow diagram of literature search and selection process.

The selection strategy of studies is summarized in Fig 1 and the quality assessment is reported in the (S1 Table).

A total of 75 studies met the inclusion criteria. Mainly the studies originated from USA, China and Germany as seen in Table 1 (S2 Table). The articles described a total of 603 cases: autopsy (66.6%), postmortem biopsy (20.9%), antemortem biopsy (9.3%), and placenta (3.2%). In most cases SARS-CoV-2 infection was diagnosed by nucleic acid testing by polymerase chain reaction (PCR) according to local protocol.

**Table 1:** Study characteristics.

Characteristics	Number (%)	Characteristics	Number (%)
**Country (n = 75)**		**Study type (n = 75)**	
United States of America	22 (29.3%)	Case reports	30
China	16 (21.3%)	Case series	45
Germany	9 (12.0%)	**Specimen source (n = 603)**	
Spain	6 (8.0%)	Autopsy	402 (66.6%)
Switzerland	6(8.0%)	Postmortem biopsy	126 (20.9%)
Italy	4 (5.3%)	Antemortem biopsy	56 (9.3%)
Brazil	3 (4.0%)	Placenta biopsy	19 (3.2%)
Belgium	2 (2.6%)	**Sample size (n = 75)**	
Austria	1 (1.3%)	≤5 cases	42 (56.0%)
Finland	1 (1.3%)	6 to ≤10 cases	15 (20.0%)
France	1 (1.3%)	11 to ≤20 cases	10 (13.3%)
Iran	1 (1.3%)	>20 cases	8 (10.7%)
Japan	1 (1.3%)		
Netherlands	1 (1.3%)		
United Kingdom	1 (1.3%)		

Patient demographics and comorbidities from the 75 studies are shown in Table 2. The median age of adult cases was 68 years (yr.) (range: 28–88), and for pediatric cases the range was 11 to 17 yr. Of 536 cases where gender was reported, 70.9% were male. The four most reported comorbidities were arterial hypertension (40.8%), diabetes mellitus (22.0%), cardiovascular disease (17.2%), and obesity (11.5%).

**Table 2:** Demographics and medical history of the COVID-19 cases.

Demographics	N = 536	% or range
Sex: male*	380/536	70.9%
Median age (Years)**	68	28–88
**Comorbidities**	**N = 546**	**%**
Hypertension	223/546	40.8
Diabetes Mellitus	120/546	22.0
Cardiovascular Disease	94/546	17.2
Obesity	63/546	11.5
Chronic Kidney disease	47/546	8.6
Tumor	44/546	8.1
Chronic Obstructive Pulmonary Disease	29/546	5.3
Dementia	16/546	2.9
Cardiac arrythmias	19/546	3.5
Dyslipedemia	19/546	3.5
Bronchial Asthma	8/546	1.5

*Data not available n = 48. Does not include the postnatal cases (n = 19)

**Does not include pediatric studies (n = 2), studies with mean age (n = 4) and data not available (n = 4)

Table 3 summarizes the radiology, laboratory results and cause of death of severe COVID-19 patients. Radiological assessment of the chest was reported in 313 (51.9%) cases showing unilateral or bilateral lung opacities (54.6%), pulmonary consolidations (32.9%) and thromboembolic events (6.1%). From studies reporting laboratory results, D-dimers were elevated in 83.2%, procalcitonin was elevated in 66.7%, markers of systemic inflammation such as C-reactive protein (CRP), ferritin, interleukin-6 (IL-6) were elevated in 91.0%, 83.3% and 74.3% of the reported cases, respectively.

**Table 3:** Imaging, clinical laboratory and cause of death of COVID-19 patients.

Imaging lung	n = 313	%
Lung opacities	171	54.6
Lung consolidation	103	32.9
Thromboembolic events	19	6.1
Lung shadows	11	3.5
Lung lesions	2	0.6
**Imaging Central Nervous System**	**n = 13**	**%**
Infarction/Ischemia	4	30.8
**Clinical laboratory**	**Abnormal/Total reported**	**%**
C-Reactive Protein	92/101	91.0
Ferritin	30/36	83.3
D-dimer	114/137	83.2
Lactate dehydrogenase	80/100	80.0
Interleukin-6	52/70	74.3
Procalcitonin	14/21	66.7
Aspartate aminotransferase	65/99	65.6
Creatinine	90/148	60.8
Alanine aminotransferase	44/88	50
Creatine Kinase	11/26	42.3
Platelet	19/102	18.6
**COVID-19 specified cause of death**	**n = 227**	**%**
Respiratory Failure	161	70.9
Multiorgan failure	25	11.0
Cardiac	24	10.6
COVID-19	16	7.0
Pneumonia	15	6.6
Septic shock	7	3.0
Pulmonary Emboli	5	2.2
Gastrointestinal	3	1.3
Cerebral hemorrhage	2	0.9
Liver cirrhosis	1	0.4
Renal failure	1	0.4
**Community deaths**	**n = 9**	

The reported main cause of death for 227 COVID-19 patients was respiratory failure (70.9%), followed by multiorgan failure (11.0%), cardiac (10.6%), COVID-19 (7.0%) and pneumonia (6.6%).

Table 4 summarizes recurrent pathologic findings in COVID-19 patients. Detailed classification of histopathologic findings in all organs and their relative frequencies are found in S3 Table in the Supplementary Appendix. DAD, the most common lung pathology was found in 315 out of 342 cases (92.1%). 94 cases (27.4%) had superimposed acute focal or diffuse bronchopneumonia. The most common abnormalities in the liver and heart were hepatitis (n = 50, 21.2%) and myocarditis (n = 24, 11.4%) respectively. Encephalitis was observed in 5 (4.6%) brains at autopsy. Vasculitis was commonly observed only in skin biopsies (25.0%).

**Table 4:** Recurrent pathologic findings by organ/organ-system.

Organ/system	Organ Specific	Microthrombi	Endothelial Injury	TE disease	Vasculitis	Inflammation*	H/D compromise
	n (%)	n (%)	n (%)	n (%)	n (%)	n (%)	n (%)
**Upper airways+**	-	-	-	-	-	33/33(100)	-
**Lung**	DAD*315/342(92.1)	132 (38.6)	61 (17.8)	47 (13.7)	10 (2.9)	21 (6.1) **	27 (7.9)
**GI**	-	2/83 (2.4)	2 (2.4)	-	-	-	15(18.1)
**Liver**	-	-	1/236 (.4)	93 (39.4)	-	59 (25.0)	114(48.3)
**Heart**	-	-	8/210 (3.8)	45(21.4)	-	37 (17.6)	-
**Kidney**	-	55/276 (19.9)	1 (0.4)	2 (0.7)	-	-	147(53.3)
**CNS**	-	20/110 (18.2)	-	9 (8.2)	-	9 (8.2)	34(30.9)
**Hem-Lymph**	-	-	-	1/136 (0.7)	-	15(11.0)	7 (5.2)
**Skin**	-	13/44 (29.5)	1 (2.3)	-	11 (25.0)	15(34.1)	-
**Placenta**	-	11/19(57.9)	-	-	-	1 (5.3)	-

GI- gastrointestinal, CNS- central nervous system, Hem-lymph- hematolymphoid, DAD- Diffuse alveolar damage, TE- thromboembolic, H/D-hemodynamic. +Trachea, pharynx, bronchial, mucosa.

* Possible cytopathic effect.

** Inflammation not related to Diffuse Alveolar Damage. Highlighted- most common abnormality by organ-system.

The presence of microthrombi was documented in the placenta (57.9%), lung (38.6%), kidney (19.9%), CNS (18.2%), and gastrointestinal (GI) tract (2.4%). Injury of endothelial cells was mostly reported in the lung (17.8%) and heart (3.8%). Thromboembolic disease was most common in the liver (39.4%) followed by the heart (21.4%) and lung (13.7%). Changes due to hemodynamic compromise such as coagulative necrosis secondary to hypoxia and/or hypoperfusion, edema and congestion were frequently seen in the kidney (53.3%), liver (48.3%), CNS (30.9%), GI tract (18.1%), lung (8%) and spleen (5.2%).

Viral particles (vp) suggestive of SARS-CoV-2 were demonstrated within specific organ cells in the trachea, lung, liver, colon, kidney, CNS either by electron microscopy (EM), immunohistochemistry (IHC) or immunofluorescence (IF) (Table 5). Among hematolymphoid tissues vp were only observed in monocytes/macrophages. In the lung and kidney vp were observed both within epithelial and endothelial cells. In the skin vp were detected only in endothelial cells. In the pancreas, heart, saphenous vein, tonsils, testes, retina, pleural effusion and placenta, testing was performed only by PCR.

**Table 5:** Detection of SARS-CoV-2 in different organs in COVID-19 patients.

System	Site	Detected (Y/N)	Method (#positive/#tested)			
			PCR	EM	IF	IHC
Upper respiratory	Pharynx	Y	35/39	NA	NA	NA
	Trachea (distal & proximal)	Y	14/14	12/12	NA	1/2
	Epiglottis	Y	1/1	NA	NA	NA
Lower respiratory	Bronchus	Y	22/23	NA	NA	NA
	Lung	Y	94/107	48/87	2/26	21/29
Gastrointestinal	Esophagus	N	0/2	NA	NA	NA
	Stomach	N	0/15	NA	NA	0/3
	Small intestine	N	0/1	NA	NA	NA
	Large intestine	Y	18/25	2/14	NA	0/1
Hepatopancreatobiliary	Liver	Y	22/40	2/2	NA	0/18
	Pancreas	Y	1/4	NA	NA	0/4
	Gallbladder	N	0/4	NA	NA	NA
Cardiovascular	Heart	Y	22/66	0/17	NA	0/5
	Saphenous vein	Y	4/12	NA	NA	NA
Hematolymphoid	Tonsils	Y	1/1	NA	NA	NA
	Lymph nodes	Y	30/30	25/25	NA	0/1
	Spleen	Y	17/ 47	2/24	NA	0/5
	Blood	Y	8/17	NA	NA	NA
	Bone marrow	N	0/3	NA	NA	0/3
Genitourinary	Kidney	Y	41/71	25/54	6/9	2/31
	Bladder	N	0/12	NA	NA	NA
	Vagina	N	0/1	NA	NA	NA
	Testes	Y	22/33	0/3	NA	NA
Endocrine	Adrenal	N	0/2	NA	NA	NA
	Thyroid	N	0/3	NA	NA	0/3
Central Nervous	Brain	Y	41/72	1/1	NA	0/22
	Retina	Y	3/14	NA	NA	NA
Skin	Skin	Y	0/6	1/1	NA	8/11
Soft tissues	Skeletal muscle	N	0/1	NA	NA	NA
Placenta		Y	1/4	NA	NA	NA
Body fluids/excretions	Pleural effusion	Y	10/10	NA	NA	NA
	CSF	N	0/11	NA	NA	NA
	Urine	N	0/2	NA	NA	NA
	Feces	N	0/1	NA	NA	NA

NA- Not Available, Y- Yes, N-No, PCR- Polymerase Chain Reaction, EM- Electron Microscopy, IF- Immunofluorescence, IHC- Immunohistochemistry

## Discussion

The findings presented on this systematic review showed demographic features, comorbidities, clinical manifestations, laboratory, and radiologic findings in line with existing literature on severe COVID-19 infection, indicating that the sample is likely representative of severe COVID-19 disease [15, 16].

### Pathologic findings

The predominant findings in fatal COVID-19 cases were DAD, coagulopathy, and hemodynamic compromise. Involvement of non-pulmonary organs was limited to parenchymal inflammation (myocarditis, hepatitis, and encephalitis), which was mostly mild. Direct viral cytopathic injury of extrapulmonary organs in general was not regarded as the cause of organ failure.

### Effects of SARS-CoV-2 in the respiratory tract

The upper respiratory tract is the initial site of viral infection; two proteins critical for the viral entry, ACE2 and TMPRSS2 are highly expressed in nasal goblet cells and ciliated cells of human airways [17]. The S protein of SARS-CoV-2 binds to ACE2 with 10-20-fold greater affinity than that of SARS-CoV-1 [18]. Male gender and smoking have been associated with increased expression of ACE2 in the lower airways and increased severity of infection [19].

The infection is thought to spread to the lower respiratory tract via secretions or leukocytes. CT imaging studies show ground-glass opacities in 93% of pre-symptomatic patients; prominent radiologic abnormalities in patients with no or minimal symptoms appear to be common [20]. Respiratory function in patients with COVID-19 infection can worsen suddenly, especially around day 9 after initiation of symptoms, leading to intensive care unit (ICU) admission. This worsening is associated with plasma elevations of acute phase reactants such as C-reactive protein, IL-6, and ESR. Procalcitonin, a parameter usually associated with systemic bacterial infection is also frequently elevated, most likely as a result of tissue injury.

Some authors have proposed that the dysregulated inflammatory response is largely restricted to the lungs; this notion is supported by the pathologic findings, which affect primarily the lung. Furthermore, inflammatory mediators such as IL-1β and IL-6 are 100- to 1000-fold higher in respiratory fluids than in serum, and RNA sequencing of bronchoalveolar lavage fluid has shown a marked increase in monocyte-derived macrophage inflammatory phenotype (enhanced transcription of STAT1, STAT2 and multiple IFN regulatory factors) [21].

In contrast with DAD occuring in other clinical contexts, in COVID-19 patients, DAD develops in non-previously critically ill individuals, more commonly in the elderly, but it can also occur in young and/or healthy individuals. In our review, the morphologic findings of COVID-19 related DAD were identical to those seen in DAD of other etiologies [22]. One study describes that the reparative angiogenesis observed in COVID-19 cases is different from that observed in Influenza A (H1N1) induced DAD: in the former the neoangiogenesis occurs predominantly through partition of existing vessels, and in the latter by proliferation (sprouting) of new vessels [23]. These results were obtained on a small sample (n = 7) and the clinical implications of such finding are uncertain.

Per our review, COVID-19 vp have been identified in all main constituents of the alveoli: pneumocytes, capillary endothelial cells, and alveolar macrophages. Endothelial cells and macrophages are major cytokine producing cells; the lung injury is presumably the result of cytopathic viral effect on pneumocytes and endothelial cells, amplified by the inflammatory response and cytokine release elicited by injured endothelial cells and activated macrophages. As the endothelial injury develops, the antithrombotic and anti-inflammatory function of the normal endothelium is lost and the balance shifts to a prothrombotic phenotype. Endothelial dysfunction leads to platelet and complement activation in addition to leukocyte accumulation in the microvasculature, as described in detail by Jackson et al. [24].

The subacute and chronic phases of DAD are primarily the reparative/scarring response to the initial injury and are characterized by an initial amplification of the inflammatory response by recruitment of acute inflammatory cells and proliferation of fibroblasts and vessels, and later by the removal of the damaged tissue by phagocytic cells, apoptosis of granulation tissue; eventually leading to restoration of the normal architecture in most cases. These processes transitorily prevent an effective gas exchange, render the lung susceptible to bacterial superinfection due to disruption of the epithelial barrier, and in a few cases, it may lead to irreversible loss of function [25].

The current management of DAD is primarily supportive, and include lower tidal volumes, optimal level of positive end-expiratory pressure, prone positioning, neuromuscular blockade, extracorporeal membrane oxygenation, corticosteroids, and antibiotic prophylaxis, all of which have been incorporated in the management of COVID-19 related DAD with modest improvements in mortality [26–28].

The acute phase of the disease is the stage most susceptible for therapeutic intervention and failure to improve during the first week of treatment is the most important negative prognostic factor [29]. Addressing the underlying cause of COVID-19 induced DAD implies effective therapy for COVID-19 infection. At present, this includes the use of systematic corticosteroids in severe and critical patients [30]. The use of anticoagulant therapy for thromboprophylaxis in hospitalized patients, to prevent the accumulation of microthrombi in the lung capillaries and to reduce the progression to systemic coagulopathy that could lead to multiorgan failure have been used with some benefit [31]. Given the known pathophysiology of DAD, the earlier therapies are initiated, the greater the benefit should be [28]. In addition, many other agents including antivirals and immunomodulators for the use in COVID-19 patients are still under investigation [31].

In the subacute phase the emphasis should shift to maintaining adequate oxygenation and hemodynamic support to prevent hypoxia/hypoperfusion/acidosis induced organ damage, adequate nutrition, and prevention of common complications such as ventilator induced lung injury, superimposed pneumonia/sepsis, and thromboembolic events while the tissues heal. Bacterial pneumonia a preventable important common complication of the subacute phase of DAD, was documented in 33% of autopsies in our review.

A prolonged clinical course frequently results in multiorgan failure [32]. In patients who survive, pulmonary function is usually restored within 6 months, although some limitations may persist up to 12 months [25]. Few patients who progressed into the chronic, scarred phase of DAD have undergone lung transplantation [33]. Extent and severity of the long-term respiratory complications in COVID-19 survivors are yet to be confirmed and will require long-term follow-up.

### Coagulopathy

In our review, the incidence of pure thromboembolic lesions at autopsy was documented in the lung, liver, and heart 14%, 39% and 21% of cases, respectively; prophylactic anticoagulation was used only in approximately 16% of fatal cases. A recent meta-analysis for the incidence of venous thromboembolism (VTE) found a similarly high incidence of 23% [34]. This high incidence of VTE is due to the connection between coagulation and inflammation, which is referred to as “thromboinflammation or immunothrombosis” to highlight the close association between inflammation and thrombosis. As Foley and Conway [35] point out, the bridge between these pathways is tissue factor (TF), which is present in high levels in lungs and in baseline conditions is expressed in the subendothelium of the vasculature. TF initiates the coagulation process by binding to activated factor VII to generate thrombin, which activates endothelial cells, platelets, leukocytes and able to propagate both microvascular thrombosis and inflammation through protease-activated receptors (PARs). The antithrombotic surface of the endothelium, maintained by nitric oxide, prostaglandin I2, antithrombin, thrombomodulin, protein C and tissue factor pathway inhibitor, becomes a procoagulant surface by expressing TF, leukocyte adhesion molecules (ICAM-1, VCAM-1), and by releasing von Willebrand factor during endothelial cell activation by thrombin. Furthermore, cytokines induce TF expression on circulating monocytes and microparticles during infection which contributes significantly to their procoagulant effect [35].

The increased expression of TF and adhesion molecules combined with release of von Willebrand factor generates microthrombi in the adjacent capillary bed, which can become systemic as the inciting event continues. Endothelial cells express ACE2 receptors [18] and infection of these cells by COVID-19 has been well documented as leading to endothelitis, defined by subendothelial accumulation of monocytes and neutrophils with detachment of endothelial cells [36]. Additionally, neutrophils at the site of infection are able to release some of their nuclear material forming a meshwork of decondensed DNA combined with histones and cytoplasmic content, called neutrophil extracellular traps (NETs) [37]. This process of NETosis is part of the innate immune process and leads to reduced blood flow and thrombus formation through activation of the intrinsic (TF-independent) coagulation pathway. Middleton et al. [38] compared NET formation in plasma of COVID-19 patients with controls and they found significantly increased plasma NET levels, as well as increased amount of soluble and cellular factors capable of inducing NET development in COVID-19 samples. Importantly, plasma NET levels were correlated with disease severity and were significantly higher in non-survivors compared with survivors and returned to normal in convalescent plasma. In addition, they detected NET-containing microthrombi in pulmonary tissue and they were able to block NET formation by adding inhibitory peptides in vitro to COVID-19 plasma. Their well-designed study supports the role of NETs in the coagulopathy seen in COVID-19 patients.

Studies have stated the frequent occurrence of disseminated intravascular coagulation (DIC) in severe COVID-19 infection associated with increased mortality. However, the laboratory findings in patients with COVID-19 infection do not fit the definition of the earlier phase of DIC, which has been designated as sepsis-induced coagulopathy (SIC) by the International Society of Thrombosis and Haemostasis (ISTH) [39]. In the majority of COVID-19 cases, fibrinogen levels are increased, coagulation tests are normal or minimally prolonged, thrombocytopenia is mild or absent, and morphologic evidence of microangiopathic hemolysis (schistocytes) is not present. The presence of a hypercoagulable state in COVID-19 patients is supported by the presence of markedly increased fibrin degradation products and D-dimers, microthrombosis in different organs and high incidence of thromboembolic events. These changes indicate that the coagulopathy of COVID-19 infection is dominated by hypercoagulability with secondary fibrinolysis. Elevated D-dimer levels of 10 to 20-fold above the upper limit of normal are described in COVID-19 patients, and an association with increased mortality is found in multiple case series [10, 11]. In our review, pathologic evidence of endothelial injury and thromboembolic disease was present in the lung in nearly all cases, while the involvement of other organs was variable, usually in the form of microthrombi or thromboembolic disease, but not hemorrhagic events. Coagulopathy in the context of lung injury is one of the cardinal aspects of DAD [40] and explained by activation of the extrinsic (TF) pathway due to tissue injury, and the intrinsic pathway by NETs. In their review of 83 COVID-19 infected patients, Fogarty et al. [41] hypothesize that a “double-hit” virally induced injury of both the alveolar cells and the capillaries due to their anatomical proximity [42].

The ISTH published interim guidance recommends prophylactic dose of low molecular weight heparin (LMWH) in all patients without active bleeding or platelet count > 25 x 10^9/L, followed by adequate laboratory monitoring [43]. While thromboprophylaxis measures are to be adopted for inpatients with COVID-19 infection [31], some groups advocate for therapeutic instead of prophylactic doses of LMWH [44–46]. The NIH recommends full dose heparin anticoagulation based on the interim results of three international trials including over 1,000 hospitalized patients with moderate COVID-19 symptoms [47] that showed decreased need for life support and improved outcomes in these patients.

Espirito Santo et al. [48] reported microvascular thrombosis *in vivo* by video capillaroscopy in 13 COVID-19 patients on mechanical ventilation while receiving LMWH. The authors concluded that microvascular thrombosis occurs systemically, and that organs with the highest capillary density are most affected. In our review microthrombi were consistent and significant only in the lungs.

### Effects of SARS-CoV-2 in non-respiratory organs

EM, IF and IHC allow the detection or direct observation of vp within organ specific cells. PCR is more sensitive than the previous methods but requires homogenized tissue samples precluding the identification of the specific source of the viral RNA. From the 603 cases included in this review, vp were observed in organ specific cells from the trachea, lung, colon, liver, lymph nodes, spleen, kidney, brain, and skin. Nonetheless, these reports would require additional validation since difficulties in differentiating subcellular structures from vp have been reported recently [49]. In the remaining organs the virus was detected by PCR. Among the hematopoietic organs tested the virus was only observed in monocytes but not lymphocytes or bone marrow cells. Hemophagocytic lymphohistiocytosis (HLH) and hemophagocytosis without HLH have been reported in a number of severe COVID19 infections [50, 51], however available literature does not support a relevant association between these disorders.

Other findings of the hematolymphoid system such as lymphocyte depletion, granulocytic hyperplasia is common in systemic infections and after steroid therapy and expected to be fully reversible [52].

In the GI tract and kidney, ACE2 is involved in amino acid homeostasis, expression of antimicrobial peptides, local innate immunity, and gut ecology [53]. While symptoms such as vomiting, diarrhea and abdominal pain were commonly documented among COVID-19 patients, histopathologic examination of gastrointestinal samples did not show significant infection related tissue damage. Two studies reported cases of ischemic necrosis of the intestine [36, 54] likely due to endothelial/hemodynamic compromise and/or coagulopathy. These changes should be reversible after the infection resolves [55].

Significant hepatic pathology was primarily related to hemodynamic changes and coagulopathy. Mild hepatitis was present in 23% of the cases. Similar abnormalities have been reported with other respiratory viruses and are expected to be fully reversible given the great regenerative capacity of the liver [56].

Significant CNS changes were primarily related to coagulopathy. Mild meningoencephalitis/encephalitis was found in ~10% of the cases; this may explain the reported neurologic symptoms/mental status changes reported in a subset of critically ill patients [57]. Rhodes et al. [58] reports of a neutrophilic microvascular endothelitis present in variable amounts and variably distributed in the examined brains, suggesting a vasculitis with autoimmune features in 10 patients. Long after recovery from the acute illness and when the virus is no longer present, patients may suffer disabling after-effects that may last for weeks or months. These include one or more of the following: fatigue, dyspnea, myalgia, joint pain, chest pain, headaches, palpitations, difficulty concentrating, short term memory loss, persistent loss of smell, and chronic stress. The pathogenesis of this “post-COVID-19 condition” is currently unknown [59], but has been hypothesized to be related to virally induced endothelial damage in the microcirculation [36]. Although recent studies report potential long-term neurological sequelae in COVID-19 patients [60], follow-up of these patients is limited and; in our review, significant infection-associated tissue CNS damage was not identified.

Dysregulation of the renin-angiotensin-system (RAS) has been documented in diabetes mellitus, arterial hypertension, and chronic lung disease, which are frequent comorbidities of severe COVID-19 patients. COVID-19 infection causes decreased ACE2 expression due internalization of the virus-receptor complex [61] decreasing its beneficial vasodilator, anti-inflammatory, antioxidant, and anti-apoptotic effects and increasing the dysregulation of RAS.

There was evidence of myocarditis in up to 11% of the cases. This may represent cytopathic viral effect as the virus has been demonstrated in the myocardium and vascular endothelium.

Renal failure has been observed in up to 15% of COVID-19 patients and correlates with severity and prognosis. While ACE2 is abundant in the brush border of proximal tubular epithelium direct infection resulting in tubulointerstitial nephritis is usually not observed. The most relevant finding in the kidney was acute tubular necrosis (ATN) present in 126 (45%) of cases. ATN is common in critically ill patients usually due to renal hypoperfusion due to hemodynamic compromise, however, a component of direct cytopathic effect cannot be entirely excluded [62]. Alternative causes for ATN include virus-induced cytokine storm which can injure the kidney directly or indirectly via effects on other organs [63]. Some authors concluded that “the most significant finding in postmortem kidneys of patients with COVID-9 infection is the absence of significant findings”. Microthrombi were observed only focally (<5% glomeruli) and only in a few cases (14%) and was not considered a significant cause of acute kidney injury [64].

Cutaneous lesions were observed mostly in younger patients with asymptomatic to mild disease and showed a broad spectrum of clinical manifestations including rashes, urticarial, vesicular, and livedoid eruptions like other viral diseases. Viral particles suggestive of SARS-CoV-2 were demonstrated in endothelial cells of the skin and all these manifestations may represent cytopathic effect. The reported skin abnormalities were not relevant for the overall outcome of the patients and should resolve after the infection subsides.

The placentas of women infected with SARS-CoV-2 show no significant increase in acute or chronic inflammatory pathology [65, 66]. Placenta changes were primarily vascular abnormalities, which are commonly found in complicated pregnancies of any cause; however increased antenatal surveillance for women with COVID-19 is prudent.

No significant findings were reported in the testes [33].

Significant advances have already been made in understanding the pathomechanisms of lethal COVID-19, and many studies have reported additional pathophysiology mechanisms of COVID-19, that need to be further explored [67, 68].

### Limitations

This review has several limitations. Case reports and case series are from different countries and institutions with different levels of complexity, some of the articles were not peer reviewed, and some studies were performed by subspecialists, which may result in lack of accuracy or overstatement of some of the reported findings. Populations from different countries with different phenotypes and, mainly, genotypes may influence the clinical manifestations, laboratory results, evolution, and the histological findings.

## Conclusion

The main pathologic/autopsy findings of severe COVID-19 infection causing fatality are DAD and changes related to coagulopathy and/or hemodynamic compromise. Knowing the pathogenesis and epidemiological, clinical, and pathological characteristics of patients with Covid-19 infection are key to identify potential targets for more effective therapies. The management guidelines developed for DAD of other etiologies translate well to COVID-19 associated DAD but need major breakthroughs given the still very high mortality of this pathology. Therapeutic interventions should be applied in the early phase of DAD, the step most amenable to intervention and should be tailored according to the timing and specific progress or development of complications. Because the coagulopathy of COVID-19 infection is different from SIC, this hypercoagulable state requires anticoagulation to improve patient outcomes.

## Supporting information

S1 Checklist Preferred reporting items for systematic reviews and meta-analyses: The PRISMA statement. (PDF)

S1 Text Literature search performed using EMBASE, MEDLINE, PubMed, MedRxiv, Lilacs and Epistemonikos. (PDF)

S1 Table Murad tool—methodological quality assessment of case reports and case series. (PDF)

S2 Table First author, country, study type, peer review, sample size, gender, and age of cases of the included studies. (PDF)

S3 Table Classification of the histopathological findings in COVID-19 cases. (PDF)
