# Supplementary material for: Pathological findings in organs and tissues of patients with COVID-19: A systematic review
Source: PLoS One. 2021 Apr 28;16(4):e0250708. doi: 10.1371/journal.pone.0250708 (PMC8081217; doi:10.1371/journal.pone.0250708)
Supplement: S1 Text — (PDF) [file pone.0250708.s002.pdf]

S1 Text. Literature search

Titles and abstracts identified from publications using EMBASE (EMBASE + MEDLINE) (94), PubMed (194), MedRxiv (8), LILACS (0) and Epistemonikos (7) were reviewed. Also included were manual searches recommended by experts and reference from other systematic reviews related to the topic.

I. **PubMed : PubMed (January - june 17, 2020): 152 (SARS Virus [mh] OR covid OR covid-19) (Autopsy [mh] OR Postmortem [tw] OR Post-Mortem [tw] OR Autopsy [tw] OR Autopsies [tw] OR Postmortem [tw] OR Biopsy [mh] OR Biopsy [tw])**

No.	Query results	Results
#1	((SARS Virus[MeSH Terms]) OR (covid)) OR (covid-19)	26,490
#2	(((((((Autopsy[MeSH Terms]) OR (Postmortem[Text Word])) OR (Post-Mortem[Text Word])) OR (Autopsy[Text Word])) OR (Autopsies[Text Word])) OR (Postmortem[Text Word])) OR (Biopsy[MeSH Terms])) OR (Biopsy[Text Word])	605,063
#3	1 and 2	194

II. **EMBASE search 17 June 2020**

No.	Query results	Results
#1	('sars coronavirus':ti,ab,kw OR covid:ti,ab,kw OR 'covid 19':ti,ab,kw OR 'novel coronavirus':ti,ab,kw) AND [1-1-2020]/sd NOT [18-6-2020]/sd	18,005
#2	(autopsy:ti,ab,kw OR autopsies:ti,ab,kw OR postmortem:ti,ab,kw OR 'post mortem':ti,ab,kw OR biopsy:ti,ab,kw OR biopsies:ti,ab,kw) AND [1-1-2020]/sd NOT [18-6-2020]/sd	25,549
#3	#1 and #2	94

EMBASE search 18 June to 4 August 2020

No.	Query results	Results
#1	('sars coronavirus':ti,ab,kw OR covid:ti,ab,kw OR 'covid 19':ti,ab,kw OR 'novel coronavirus':ti,ab,kw)	20675
#2	(autopsy:ti,ab,kw OR autopsies:ti,ab,kw OR postmortem:ti,ab,kw OR 'post mortem':ti,ab,kw OR biopsy:ti,ab,kw OR biopsies:ti,ab,kw)	3988
#3	#1 and #2	148

III. **MEDRxiv (June 17): 46**

COVID-19 database, Refined by Pathology and Forensic Medicine – 8 selected

IV. **LILACS – BIREME (June 17**): 9 references: 0 selected

V. **Epistemonikos (June 17):** 16 references: 7 selected
