# Supplementary material for: Pathological findings in organs and tissues of patients with COVID-19: A systematic review
Source: PLoS One. 2021 Apr 28;16(4):e0250708. doi: 10.1371/journal.pone.0250708 (PMC8081217; doi:10.1371/journal.pone.0250708)
Supplement: S1 Table — (PDF) [file pone.0250708.s003.pdf]

S1 Table: Murad tool - methodological quality assessment of case reports and case series

Ref	First Author	Does the patient(s) represent(s) the whole experience of the investigator (centre) or is the selection method unclear to the extent that other patients with similar presentation may not have been reported?	Was the exposure adequately ascertained?	Was the outcome adequately ascertained?	Were other alternative causes that may explain the observation ruled out?	Was follow-up long enough for outcomes to occur?	Is the case(s) described with sufficient details to allow other investigators to replicate the research or to allow practitioners make inferences related to their own practice?
1	Remmelink M	Yes	Yes	Yes	Yes	No	Yes
2	Prilutskiy A	No	Yes	Yes	Yes	Yes	Yes
3	Lax SF	Yes	Yes	Yes	Yes	Yes	Yes
4	De Voeght A	Unclear	Yes	Yes	Yes	Yes	Yes
5	Yao XH	Unclear	Yes	Yes	Yes	Yes	Yes
6	Liu Q	No	Yes	Yes	Yes	Yes	Yes

7	Su H	Yes	Yes	Yes	Yes	Yes	Yes
8	Yang M	Yes	Yes	Yes	Yes	Yes	Yes
9	Fitzek A	No	Yes	Yes	Yes	Yes	Yes
10	Wichmann D	Yes	Yes	Yes	Yes	Yes	Yes
11	von Weyhern CH	Unclear	Yes	Yes	Yes	Yes	Yes
12	Löffler KU	No	Yes	Yes	Yes	Yes	Yes
13	Schaller T	Yes	Yes	Yes	Yes	No	No
14	Ackermann M	Unclear	Yes	Yes	Yes	Yes	Yes
15	Sonzogni A	Yes	Yes	Yes	Yes	Yes	No
16	Carsana L	Yes	Yes	Yes	Yes	Yes	Yes
17	Previtali G	Yes	Yes	Yes	Yes	Yes	Yes
18	Adachi T	No	Yes	Yes	Yes	Yes	Yes
19	Conde PN	No	Yes	Yes	Yes	Yes	Yes
20	Autopsias P	No	Yes	Yes	Yes	Yes	No
21	Suess C	No	Yes	Yes	Yes	Yes	No
22	Schweitzer W	No	Yes	Yes	Yes	No	Yes
23	Aguiar D	No	Yes	Yes	Yes	Yes	Yes

24	Menter T	Yes	Yes	Yes	Yes	Yes	Yes
25	Barton LM	No	Yes	Yes	Yes	Yes	Yes
26	Konopka KE	No	Yes	Yes	Yes	Yes	Yes
27	Bradley BT	Yes	Yes	Yes	Yes	Yes	Yes
28	Sekulic M	No	Yes	Yes	Yes	Yes	Yes
29	Solomon IH	Yes	Yes	Yes	Yes	Yes	Yes
30	Reichard RR	No	Yes	Yes	Yes	No	No
31	Bryce C	Yes	Yes	Yes	Yes	Yes	Yes
32	Konopka KE	No	Yes	Yes	Yes	Yes	No
33	Fox SE	Unclear	Yes	Yes	Yes	Yes	Yes
34	Farkash EA	No	Yes	Yes	Yes	Yes	Yes
35	Paniz-Mondolfi A	No	Yes	Yes	Yes	Yes	Yes
36	Craver R	No	Yes	Yes	Yes	Yes	Yes
37	Yan L	No	Yes	Yes	Yes	Yes	Yes
38	Magro C	No	Yes	Yes	Yes	Yes	Yes
39	Varga Z	No	Yes	Yes	Yes	Yes	No
40	Mahe A	No	Yes	Yes	Yes	Yes	Yes

41	Escher F	Yes	Yes	Yes	Yes	Yes	Yes
42	Rossi GM	No	Yes	Yes	Yes	Yes	Yes
43	Llamas-Velasco M	No	Yes	Yes	Yes	Yes	Yes
44	Colmenero I	Unclear	Yes	Yes	Yes	Yes	No
45	Reymundo A	Yes	Yes	Yes	Yes	No	Yes
46	Tian S	No	Unclear	Yes	Yes	Yes	No
47	Dolhnikoff M	Yes	Yes	Yes	Yes	Yes	No
48	Chen S	No	Yes	Yes	Yes	Yes	Yes
49	Baud D	No	Yes	Yes	Yes	Yes	No
50	Shanes ED	Yes	Yes	Yes	Yes	Yes	Yes
51	Nagashima S	Unclear	Yes	Yes	Yes	Yes	Yes
52	Li S	Yes	Yes	Yes	Yes	Yes	Yes
53	Shao C	No	Yes	Yes	Yes	Yes	Yes
54	Zhang H	No	Yes	Yes	Yes	Yes	No
55	Wu J	No	Yes	Yes	Yes	Yes	No
56	Xu X	No	Yes	Yes	Yes	Yes	No
57	Xu Z	No	Yes	Yes	Yes	Yes	No

58	Tian S	No	Yes	Yes	Yes	Yes	Yes
59	Wang Y	No	Yes	Yes	Yes	Yes	Yes
60	Copin MC	No	Yes	Yes	Yes	Yes	No
61	Casagrande M	Yes	Yes	Yes	Yes	Yes	Yes
62	Su H	Yes	Yes	Yes	Yes	Yes	Yes
63	Santana MF	No	Yes	Yes	Yes	Yes	No
64	Kudose S	Yes	Yes	Yes	Yes	Yes	Yes
65	Golmai P	Yes	Yes	Yes	Yes	Yes	Yes
66	Flikweert AW	Yes	Yes	Yes	Yes	Yes	Yes
67	Sharma P	Yes	Yes	Yes	Yes	Yes	Yes
68	Youd E	Yes	Unclear	Yes	Yes	Yes	Yes
69	Magoon S	No	Yes	Yes	Yes	Yes	Yes
70	Rapkiewicz AV	Unclear	Yes	Yes	Yes	Yes	Yes
71	Beigmohammadi MT	No	Yes	Yes	Yes	Yes	Yes
72	Wang C	No	Yes	Yes	Yes	Yes	Yes
73	Prieto-Pérez L	Yes	Yes	Yes	Yes	Yes	Yes

74	Sauter JL	Unclear	Yes	Yes	Yes	Yes	Yes
75	Kantonen J	No	Yes	Yes	Yes	Yes	Yes
