# Supplementary material for: Pathological findings in organs and tissues of patients with COVID-19: A systematic review
Source: PLoS One. 2021 Apr 28;16(4):e0250708. doi: 10.1371/journal.pone.0250708 (PMC8081217; doi:10.1371/journal.pone.0250708)
Supplement: S2 Table — (PDF) [file pone.0250708.s004.pdf]

S2 Table: First author, country, study type, peer review, sample size, gender and age of the included studies

Ref.	First Author	Country	Title	Peer reviewed Yes/No	Study type	Sample size	Specimen source	M, F	Age in years median (range)
1	Remmelink M	Belgium	Unspecific post-mortem findings despite multiorgan viral spread in COVID-19 patients	Yes	Case series	17	Autopsy	12,5	72 (62-77)
2	Prilutskiy A	USA	SARS-CoV-2 Infection associated hemophagocytic lymphohistiocytosis: An autopsy series with clinical and laboratory correlation.	Yes	Case series	4	Autopsy	3,1	72 (64-91)
3	Lax SF	Austria	Pulmonary arterial thrombosis in COVID-19 with fatal outcome: Results from a prospective, single-center, clinicopathologic case series	Yes	Case series	11	Autopsy	8,3	81(66-91)
4	De Voeght A	Belgium	Thrombotic microvascular injury is not mediated by thrombotic microangiopathy despite systemic complement activation in Covid-19 patients	No	Case series	2	Autopsy	NA	NA
5	Yao XH	China	A pathological report of three COVID-19 cases by minimal invasive autopsies	Yes	Case reports	3	Autopsy	2,1	69 (63-79)
6	Liu Q	China	Gross examination report of a COVID-19 death autopsy	Yes	Case report	1	Autopsy	1,0	85
7	Su H	China	Renal histopathological analysis of 26 postmortem findings of patients with COVID-19 in China	Yes	Case series	26	Autopsy	19,7	69 (39-87)
8	Yang M	China	Pathological findings in the testes of COVID-19 patients: Clinical implications	Yes	Case series	11	Autopsy	11,0	66(42-87)

9	Fitzek A	Germany	Evidence for systematic autopsies in COVID-19 positive deceased: Case report of the first German investigated COVID-19 death	Yes	Case report	1	Autopsy	1,0	59
10	Wichmann D	Germany	Autopsy Findings and Venous Thromboembolism in Patients with COVID-19	Yes	Case series	12	Autopsy	9,3	73 (52-87)
11	von Weyhern CH	Germany	Early evidence of pronounced brain involvement in fatal COVID-19 outcomes	Yes	Case series	6	Autopsy	4,2	69 (58-82)
12	Löffler KU	Germany	Ocular post-mortem findings in patients having died from COVID-19	Yes	Case series	3	Autopsy	NA	88 (67-95)
13	Schaller T	Germany	Postmortem Examination of Patients With COVID-19	Yes	Case series	10	Autopsy	7,3	79 (64-90)
14	Ackermann M	Germany	Pulmonary Vascular Endothelialitis, Thrombosis, and Angiogenesis in Covid-19	Yes	Case series	7	Autopsy	5,2	78 (66-96)
15	Sonzogni A	Italy	Liver and COVID 19 infection: a very preliminary lesson learnt from histological post-mortem findings in 48 patients	No	Case series	48	Autopsy	35,13	71. (32-87)
16	Carsana L	Italy	Pulmonary post-mortem findings in a series of COVID-19 cases from Northern Italy	Yes	Case series	38	Autopsy	33,5	Mean 69 (32-86)
17	Previtali G	Italy	The pathogenesis of thromboembolic disease in covid-19 patients: Could be a catastrophic antiphospholipid syndrome?	Yes	Case series	35	Autopsy	26,9	73 (52-82)
18	Adachi T	Japan	Clinicopathologic and Immunohistochemical Findings from Autopsy of Patient with COVID-19, Japan	Yes	Case report	1	Autopsy	0,1	84 (84)
19	Conde PN	Spain	Autopsy findings from the first known death from Severe Acute Respiratory Syndrome SARS-CoV-2 in Spain	Yes	Case report	1	Autopsy	1,0	69
20	Autopsias P	Spain	The first COVID-19 autopsy in Spain performed during the early stages of the pandemic	Yes	Case report	1	Autopsy	1,0	54

21	Suess C	Switzerland	Gross and histopathological pulmonary findings in a COVID-19 associated death during self-isolation	Yes	Case report	1	Autopsy	1,0	59
22	Schweitzer W	Switzerland	Implications for forensic death investigations from first Swiss post-mortem CT in a case of non-hospital treatment with COVID-19	Yes	Case report	1	Autopsy	1,0	50
23	Aguiar D	Switzerland	Inside the lungs of COVID-19 disease	Yes	Case report	1	Autopsy	0,1	31
24	Menter T	Switzerland	Post-mortem examination of COVID19 patients reveals diffuse alveolar damage with severe capillary congestion and variegated findings of lungs and other organs suggesting vascular dysfunction	Yes	Case series	21	Autopsy	17,4	mean 76 (53-96)
25	Barton LM	USA	COVID-19 Autopsies, Oklahoma, USA	Yes	Case reports	2	Autopsy	2,0	60 (42-77)
26	Konopka KE	USA	Diffuse Alveolar Damage (DAD) from Coronavirus Disease 2019 Infection is Morphologically Indistinguishable from Other Causes of DAD	Yes	Case series	8	Autopsy	5,3	52 (37-75)
27	Bradley BT	USA	Histopathology and ultrastructural findings of fatal COVID-19 infections in Washington State: a case series	Yes	Case series	14	Autopsy	6,8	73.5 (42-84)
28	Sekulic M	USA	Molecular Detection of SARS-CoV-2 Infection in FFPE Samples and Histopathologic Findings in Fatal SARS-CoV-2 Cases	Yes	Case reports	2	Autopsy	2,0	67.5 (54-81)
29	Solomon IH	USA	Neuropathological Features of Covid-19	Yes	Case series	18	Autopsy	14,4	62 (48-90)
30	Reichard RR	USA	Neuropathology of COVID-19: a spectrum of vascular and acute disseminated encephalomyelitis (ADEM)-like pathology	Yes	Case report	1	Autopsy	1,0	71

31	Bryce C	USA	Pathophysiology of SARS-CoV-2: targeting of endothelial cells renders a complex disease with thrombotic microangiopathy and aberrant immune response. The Mount Sinai COVID-19 autopsy experience	No	Case series	25	Autopsy	NA	NA
32	Konopka KE	USA	Postmortem Lung Findings in an Asthmatic Patient with Coronavirus Disease 2019	Yes	Case report	1	Autopsy	1,0	37
33	Fox SE	USA	Pulmonary and cardiac pathology in African American patients with COVID-19: an autopsy series from New Orleans	Yes	Case series	10	Autopsy	NA	64 (44-78)
34	Farkash EA	USA	Ultrastructural Evidence for Direct Renal Infection with SARS-CoV-2	Yes	Case report	1	Autopsy	1,0	53
35	Paniz-Mondolfi A	USA	Central nervous system involvement by severe acute respiratory syndrome coronavirus-2 (SARS-CoV-2)	Yes	Case report	1	Autopsy	1,0	74
36	Craver R	USA	Fatal Eosinophilic Myocarditis in a Healthy 17-Year-Old Male with Severe Acute Respiratory Syndrome Coronavirus 2 (SARS-CoV-2)	Yes	Case report	1	Autopsy	1,0	17
37	Yan L	USA	Autopsy Report with Clinical Pathological Correlation	Yes	Case report	1	Autopsy	0,1	44
38	Magro C	USA	Complement associated microvascular injury and thrombosis in the pathogenesis of severe COVID-19 infection: A report of five cases	Yes	Case reports	5	Autopsy (2/5), AMB (3/5)	3,2	62 (32- 73)
39	Varga Z	Switzerland	Endothelial cell infection and endotheliitis in COVID-19	Yes	Case reports	3	Autopsy (2/3), AMB (1/3)	2,1	69 (58-71)
40	Mahe A	France	Histology of skin lesions establishes that the vesicular rash associated with COVID-19 is not "varicella-like"	Yes	Case series	3	AMB	1,2	55 (50-55)
41	Escher F	Germany	Detection of viral SARS-CoV-2 genomes and histopathological changes in endomyocardial biopsies	Yes	Case series	5	AMB	4,1	48 (36-62)

42	Rossi GM	Italy	Kidney biopsy findings in a critically ill COVID-19 patient with dialysis-dependent acute kidney injury: a case against "SARS-CoV-2 nephropathy"	Yes	Case report	1	AMB	1,0	49
43	Llamas-Velasco M	Spain	Thrombotic occlusive vasculopathy in skin biopsy from a livedoid lesion of a COVID-19 patient	Yes	Case report	1	AMB	1,0	61
44	Colmenero I	Spain	SARS-CoV-2 endothelial infection causes COVID-19 chilblains: histopathological, immunohistochemical and ultrastructural study of 7 paediatric cases	Yes	Case series	7	AMB	4,3	15 (11-17)
45	Reymundo A	Spain	Clinical and histological characterization of late appearance maculopapular eruptions in association with the coronavirus disease 2019. A case series of seven patients	Yes	Case series	4	AMB	1,3	62.5 (57-67)
46	Tian S	China	Pulmonary pathology of early-phase 2019 Novel Coronavirus (COVID-19) Pneumonia in two patients with lung cancer	Yes	Case reports	2	AMB	1,1	78.5 (73-84)
47	Dolhnikoff M	Brazil	Pathological evidence of pulmonary thrombotic phenomena in severe COVID-19	Yes	Case series	10	PMB	5,5	mean 67.8 (33-83)
48	Chen S	China	Pregnant women with new coronavirus infection: a clinical characteristic and placental pathological analysis of three cases	Yes	Case series	3	Placenta	0,3	32 (23-34)
49	Baud D	Switzerland	Second-Trimester Miscarriage in a pregnant woman with SARS-CoV-2 Infection	Yes	Case report	1	Placenta	0,1	28
50	Shanes ED	USA	Placental pathology in COVID-19	Yes	Case series	15	Placenta	0,15	33 (23-41)
51	Nagashima S	Brazil	The Endothelial Dysfunction and Pyroptosis Driving the SARS-CoV-2 Immune-Thrombosis	Yes	Case series	6	PMB	4,2	80.5 (53-87)
52	Li S	China	Clinical and pathological investigation of severe COVID-19 patients	Yes	Case series	2	PMB	NA	NA

53	Shao C	China	Evolution of severe acute respiratory syndrome coronavirus 2 RNA test results in a patient with fatal coronavirus disease 2019: a case report	Yes	Case report	1	PMB	1,0	65
54	Zhang H	China	Histopathologic changes and SARS–CoV-2 immunostaining in the lung of a patient with COVID-19	Yes	Case report	1	PMB	1,0	72
55	Wu J	China	Pathological changes of fatal coronavirus disease 2019 (COVID-19) in the lungs: report of 10 cases by postmortem needle autopsy	Yes	Case series	10	PMB	7,3	73.5 (39-87)
56	Xu X	China	Pathological changes of the spleen in ten patients with coronavirus disease 2019(COVID-19) by postmortem needle autopsy	Yes	Case series	10	PMB	7,3	73.5 (39-87)
57	Xu Z	China	Pathological findings of COVID-19 associated with acute respiratory distress syndrome	Yes	Case report	1	PMB	1,0	50
58	Tian S	China	Pathological study of the 2019 novel coronavirus disease (COVID-19) through postmortem core biopsies	Yes	Case series	4	PMB	3,1	76 (59-81)
59	Wang Y	China	SARS-CoV-2 infection of the liver directly contributes to hepatic impairment in patients with COVID-19	Yes	Case reports	2	PMB	1,1	64.5 (50-79)
60	Copin MC	Germany	Time to consider histologic pattern of lung injury to treat critically ill patients with COVID-19 infection	Yes	Case series	6	PMB	NA	NA
61	Casagrande M	Germany	Detection of SARS-CoV-2 in Human Retinal Biopsies of Deceased COVID-19 Patients	Yes	Case series	14	PMB	10,4	mean 77
62	Su H	China	Renal histopathological analysis of 26 postmortem findings of patients with COVID-19 in China	Yes	Case series	26	Autopsy	19,7	68 (39-87)
63	Santana MF	Brazil	Confirmed invasive pulmonary aspergillosis and COVID-19: The value of postmortem findings to support antemortem management	Yes	Case report	1	Autopsy	1,0	71
64	Kudose S	USA	Kidney Biopsy Findings in Patients with COVID-19	Yes	Case series	17	AMB	12,5	54 (22-72)

65	Golmai P	USA	Histopathologic and Ultrastructural Findings in Postmortem Kidney Biopsy Material in 12 Patients with AKI and COVID-19	Yes	Case series	12	PMB	10,2	75 (49-92)
66	Flikweert AW	Netherlands	Late histopathologic characteristics of critically ill COVID-19 patients: Different phenotypes without evidence of invasive aspergillosis, a case series	Yes	Case series	7	PMB	5,2	74 (58-83)
67	Sharma P	USA	COVID-19-Associated Kidney Injury: A Case Series of Kidney Biopsy Findings	Yes	Case series	10	AMB	5,5	64 (45-77)
68	Youd E	UK	COVID-19 autopsy in people who died in community settings: the first series	Yes	Case series	3	Autopsy	1,2	86 (73-88)
69	Magoon S	USA	COVID-19–Related Glomerulopathy: A Report of 2 Cases	Yes	Case reports	2	AMB	1,1	42 (28-56)
70	Rapkiewicz AV	USA	Megakaryocytes and platelet-fibrin thrombi characterize multi-organ thrombosis at autopsy in COVID-19: A case series	Yes	Case series	7	Autopsy	3,4	60 (44-65)
71	Beigmohammadi MT	Iran	Pathological findings of postmortem biopsies from lung, heart, and liver of 7 deceased COVID-19 patients	Yes	Case series	7	PMB	5,2	72 (46-84)
72	Wang C	China	Alveolar macrophage dysfunction and cytokine storm in the pathogenesis of two severe COVID-19 patients	Yes	Case reports	2	Autopsy	1,1	58 (53-62)
73	Prieto-Pérez L	Spain	Histiocytic hyperplasia with hemophagocytosis and acute alveolar damage in COVID-19 infection	Yes	Case series	33	PMB	21,12	79 (53-98)
74	Sauter JL	USA	Insights into pathogenesis of fatal COVID-19 pneumonia from histopathology with immunohistochemical and viral RNA studies	Yes	Case series	8	Autopsy	4,4	58 (39-65)
75	Kantonen J	Finland	Neuropathologic features of four autopsied COVID-19 patients	Yes	Case series	4	Autopsy	3,1	72 (38-90)

AMB: Antemortem biopsy; PMB: Postmortem biopsy
