# Supplementary material for: Pathological findings in organs and tissues of patients with COVID-19: A systematic review
Source: PLoS One. 2021 Apr 28;16(4):e0250708. doi: 10.1371/journal.pone.0250708 (PMC8081217; doi:10.1371/journal.pone.0250708)
Supplement: S3 Table — (PDF) [file pone.0250708.s005.pdf]

S3 Table: Classification of the histopathological findings in COVID-19 cases

Organ	Taxonomy	Histopathological findings	Prevalence
Lung	Diffuse Alveolar Damage	• DAD any phase (acute, organizing, fibrotic)<br>• Hyaline membranes<br>• Alveolar fibrin plugs<br>• Loss of pneumocytes<br>• Reactive pneumocytes and syncytial cells<br>• Vesicular nuclei with prominent nucleoli<br>• Type 2 pneumocyte hyperplasia<br>• Microthrombi<br>• Increased intra alveolar megakaryocytes	315
	Coagulopathy associated abnormalities	• Microthrombi	173
	Endothelial injury	• Endotheliitis<br>• Endothelial necrosis	61
	Thromboembolic disease	• Medium/large vessel thromboembolism<br>• Pulmonary infarction	47
	Vasculitis	• Vasculitis<br>• Capillaritis<br>• Diffuse alveolar hemorrhage<br>• Alveolar hemorrhage<br>• Pulmonary hemorrhage	10
	Possible cytopathic effect encompasses	• Atypical polymorphous pneumocytes<br>• Apoptotic bodies<br>• Intranuclear inclusions	21
	Serositis	• Acute pleuritis	1
	Non-specific lung scarring	• Proliferation myofibroblastic<br>• Organizing pneumonia pattern<br>• Interstitial fibrosis<br>• Lymphocyte infiltration<br>• Prominent lymphoid infiltrates<br>• Lymphocytes CD4/CD8<br>• Interstitial T-cell reaction<br>• Angiogenesis/Capillary proliferation<br>• Squamous metaplasia	103
	Changes due to hemodynamic compromise	• Pulmonary capillary congestion<br>• Intra alveolar edema	27
	Complications	• Bacterial pneumonia	94
		• Hemophagocytosis	1
	Pre-existent or non-significant conditions	• Emphysema<br>• Amyloidosis of pulmonary vessels<br>• Mononuclear macrophage	29
Upper airways inflammation	Inflammation	• Pharyngeal hyperemia<br>• Tracheitis<br>• Edema bronchial mucosa	33
Liver	Possibly pre-existent parenchymal abnormalities encompass	• Steatosis = fatty changes = NASH<br>• Cholestasis, ductal proliferation	93
	Endothelial injury	• Endotheliitis	1
	Thromboembolic disease	• Venous outflow obstruction<br>• Post vein phlebosclerosis (portal ?)<br>• Thrombosis	93

	Possible cytopathic effect encompasses	• Apoptotic hepatocytes	2
		• Hepatitis: Portal inflammation, periportal and lobular T-cell reaction, Lymphocytic infiltrate, Lobular inflammation, Kupffer cell proliferation	50
	Abnormalities secondary to hemodynamic compromise	• Hepatomegaly<br>• Edema/congestion<br>• Shock liver (common perimortem terminal event)<br>• Aberrant portal vessels<br>• Periportal abnormal vessels	114
	Non-specific scarring	Fibrosis NOS	60
	Pre-existent or non-significant conditions :	Cirrhosis, Gall bladder – enlarged, Liver cyst	10
**CARDIOVASCULAR**	Endothelial injury	• Endotheliitis	8
	Thromboembolic disease	• Thrombosis	45
	Possible cytopathic effect	• Cardiomyocyte necrosis<br>• Apoptotic bodies<br>• Myocarditis (24)- Interstitial infiltration, Lymphocytic and Eosinophilic myocarditis	37
	Serositis	• Epicarditis<br>• Epicardial inflammation<br>• Pericardial edema<br>• Pericardial effusion	4
	Complications	• Cardiomyopathy (if not pre-existent)<br>• Hemophagocytosis	5
	Pre-existent cardiovascular disease	• Myocardial/myocyte hypertrophy<br>• Atherosclerosis<br>• Ischemic cardiomyopathy<br>• MI<br>• Myocardial/interstitial fibrosis<br>• Cardiomegaly	115
	Pre-existent or non-significant conditions	• Amyloidosis	8
KIDNEY/GENITOURINARY	Endothelial injury	• Endotheliitis	1
	Thromboembolic disease	• Artery thrombus<br>• Thromboemboli	2
	Possible viral effect	• Glomerulopathies (if not pre-existent) Includes FSGS, MNS, mesangial expansion	9
	Abnormalities secondary to hemodynamic compromise	• Congestion<br>• Shock kidney<br>• Congestion/medullary edema<br>• Acute tubular injury	147
	Coagulopathy associated abnormalities	• DIC<br>• Capillary thrombus<br>• Segmental fibrin thrombus	55
	Non-specific scarring	• Granular surface/scarring<br>• Cortical fibrosis	24
	Pre-existent vascular disease	• Arterionephrosclerosis<br>• Benign nephrosclerosis	76
	Pre-existent or non-significant conditions	• Cysts<br>• Testicular atrophy<br>• Amyloidosis	47

		• Pigment casts<br>• Glomerulopathies (if present before infection) (FSGS, MNS, mesangial expansion)<br>• Prostate thrombosis<br>• Benign prostatic hyperplasia<br>• Hypospermatogenesis<br>• Mild peritubular hyalinization	
Central Nervous System	Abnormalities secondary to hemodynamic compromise	• Cerebral edema/congestion<br>• Hypoxic injury<br>• Anoxic injury	34
	Coagulopathy associated abnormalities	• Hemorrhage<br>• Microthrombi	20
	Thromboembolic disease	• Infarcts (if recent)	9
	Inflammation (Possible cytopathic effect)	• Leptomeningeal inflammation<br>• Perivascular inflammation<br>• Encephalitis: 5<br>• Lymphocytic meningitis	9
	Pre-existent vascular disease:	• Cerebral chronic infarcts<br>• Atherosclerosis	18
	Pre-existent or non-significant conditions	• Subdural hematoma<br>• Cerebral cortex atrophy<br>• Pale substantia nigra<br>• Focal spongiosis<br>• Lewy body disease<br>• Alzheimer's disease<br>• Microglial nodule<br>• Neuronal cell loss<br>• Axonal degeneration<br>• Residual anaplastic astrocytoma<br>• Cytolysis	35
GASTROINTESTINAL	Endothelial injury	• Endotheliitis	2
	Coagulopathy associated abnormalities	• Hemorrhage	3
	Abnormalities secondary to hemodynamic compromise or thromboembolic disease	• Mesenteric necrosis<br>• Ischemic enterocolitis<br>• Pancreatitis	15
	Possible cytopathic effect	• Apoptotic bodies	2
	Pre-existent or non-significant conditions	• Esophagitis<br>• Diverticulosis<br>• Chronic gastritis	2
HEMATOLYMPHOID	Possible cytopathic effect	• Increased plasma blasts<br>• Apoptosis	15
	Abnormalities secondary to hemodynamic compromise	• Congestion<br>• Infarction	7
	Thromboembolic disease	• Arteriolar thrombosis	1
	Non-specific changes secondary to systemic infection	• Decreased myelopoiesis<br>• Lymphadenopathy<br>• Splenomegaly<br>• Bone marrow hyperplasia<br>• Left-shifted myelopoiesis<br>• Left-shifted granulopoiesis<br>• Splenitis<br>• Plasma cells in spleen	41
	Changes likely due to steroid therapy	• Lymphoid depletion<br>• Reduced white pulp	29
	Complications	• Hemophagocytosis	21

SKIN	Endothelial injury	• Endotheliitis	1
	Coagulopathy associated abnormalities	• Petechiae<br>• Dermal necrosis<br>• Dermo-hypodermal/superficial thrombi<br>• Deep dermis thrombi	12
	Coagulopathy associated abnormalities	• Thrombogenic vasculopathy	1
	Vasculitis	• Livedo<br>• Purpura<br>• Subcutaneous lymphocytic vasculitis<br>• Lymphocytic infiltration of vessels	11
	Possible cytopathic effect	• Intranuclear viral inclusions: 1<br>• Multinucleated cells: 1<br>• Intraepidermal vesicle<br>• Dyskeratosis<br>• Necrotic keratinocytes	7
	Non-specific findings associated with skin inflammation (dermatitis)	• Exocytosis<br>• Spongiosis<br>• Acantholysis<br>• Vesicles<br>• Suprabasal clefts<br>• Dermal edema<br>• Vascular ectasia<br>• Dermal mucin<br>• Dermal eosinophils<br>• Superficial dermal inflammatory infiltrates<br>• Perivascular deep inflammation<br>• Peri-eccrine inflammation<br>• Lymphocytic panniculitis<br>• Parakeratosis	15
PLACENTA	Possibly associated with coagulopathy	• Infarcts<br>• Fibrin deposition<br>• Villous agglutination	11
	Inflammation	• Inflammatory infiltrates<br>• Funisitis	1
	Common non-specific changes of varied etiology (e.g. hypertension, diabetes, smoking etc.)	• accelerated villous maturation<br>• Hypertrophy of membrane arterioles<br>• Decidual arteriopathy<br>• Maternal vascular malperfusion<br>• Necrosis of maternal vessels	15
	Pre-existent/unrelated findings	• Chorionic hemangioma	1
